# Andrographolide Inhibits Expression of NLPR3 Inflammasome in Canine Mononuclear Leukocytes

**DOI:** 10.3390/ani14142036

**Published:** 2024-07-11

**Authors:** Alejandro Albornoz, Bibiana Pardo, Sofia Apaoblaza, Claudio Henriquez, Javier Ojeda, Benjamín Uberti, Juan Hancke, Rafael A. Burgos, Gabriel Moran

**Affiliations:** 1 Laboratory of Inflammation Pharmacology and Immunometabolism, Institute of Pharmacology and Morphophysiology, Faculty of Veterinary Sciences, Universidad Austral de Chile, Valdivia 5090000, Chile; alejandroalbornoz.p@gmail.com (A.A.); bibianapardo@gmail.com (B.P.); sofyapa@gmail.com (S.A.); claudio.henriquez@uach.cl (C.H.); 2 Graduate School, Faculty of Veterinary Sciences, Universidad Austral de Chile, Valdivia 5090000, Chile; 3 Institute of Veterinary Clinical Sciences, Faculty of Veterinary Sciences, Universidad Austral de Chile, Valdivia 5090000, Chile; javierojeda@uach.cl (J.O.); benjamin.uberti@uach.cl (B.U.); 4 HP Animal Health, Valdivia 5090000, Chile; jhanckeo@gmail.com

**Keywords:** andrographolide, inflammasome, NLRP3, leukocytes, canine

## Abstract

**Simple Summary:**

Inflammasomes are intricate protein complexes initiating caspase-1 activation, resulting in the release of inflammatory cytokines and pyroptosis. In canines, the conserved NLRP3 gene and observed caspase-1/4 activity implicate the NLRP3 inflammasome in specific inflammatory ailments. Andrographolide, sourced from Andrographis paniculate, exhibits diverse biological effects, notably antioxidant and anti-inflammatory properties, with clinical studies hinting at its therapeutic potential across various diseases. This study seeks to explore andrographolide’s impact on gene expression linked to the NLRP3 inflammasome and cytokines in canine blood cells. The findings indicate that andrographolide diminishes the expression of NLRP3, caspase-1/4, IL-1β, IL-18, and proinflammatory cytokines, alongside reducing IL-1β secretion. This underscores andrographolide’s ability to impede inflammasome activation, thereby mitigating inflammation. Nonetheless, further research is imperative to comprehensively unravel its mechanisms and therapeutic efficacy concerning canine inflammatory conditions.

**Abstract:**

Inflammasomes are multiprotein complexes that trigger processes through caspase-1 activation, leading to the maturation of proinflammatory cytokines, such as IL-1β and IL-18. The gene encoding the inflammasome stimulatory protein NLRP3 is conserved in canines. Caspase-1/4 homologues have been identified in multiple carnivores, including canines, and caspase-1 activity has been shown in humans. The NLRP3 inflammasome has also been described in some canine inflammatory diseases. Andrographolide, a labdane diterpene, is the principal active ingredient in the herb Andrographis paniculate. The objective of this study was to determine the effect of andrographolide on the gene expression of the components of the NLRP3 inflammasome, proinflammatory cytokines, and IL-1β secretion in canine peripheral blood mononuclear cells. For this, MTT assays and real-time PCR were employed to assess the cytotoxicity and gene expression. Further, an ELISA test was performed to measure the IL-1β concentration. The findings reveal that andrographolide significantly reduces the expression of NLRP3, caspase-1/4, IL-1β, and IL-18. Additionally, it decreases the secretion of IL-1β and other proinflammatory cytokines, including IL-6, IL-8, and TNF-α. The results show that andrographolide decreases the expression of NLRP3, caspase-1/4, IL-1β, and IL-18. Andrographolide also reduces proinflammatory cytokines expression, and decreases IL-1β secretion. This indicates that andrographolide can interfere with the activation and function of the inflammasome, resulting in a decrease in the inflammatory response in canines. Research in this area is still budding, and more studies are necessary to fully understand andrographolide’s mechanisms of action and its therapeutic potential in relation to the NLRP3 inflammasome in dogs.

## 1. Introduction

Innate immunity is a quick and highly conserved biological defense against cell damage by pathogenic infections, cell stress, and injury. The innate response is largely mediated by pattern recognition receptors (PRRs), germ-line-encoded proteins that recognize conserved microbial motifs (Pathogen-Associated Molecular Patterns (PAMPs)), and endogenous danger signals (Damage-Associated Molecular Patterns (DAMPs)) [1,2]. NOD-like receptors (NLRs) are amongst the most important PRRs; 22 NLRs have been identified in humans [3]. NLRP3 is a well-characterized cytosolic NOD-like PRR which can detect microbial motifs, endogenous danger, and stress signals. NLRP3 activation, in turn, leads to the formation of a signaling complex called the inflammasome, a cytosolic multiprotein platform for caspase-1 (Casp-1) activation, which in turn induces proinflammatory cytokines IL-1β and IL-18 [4]. A second signal induces the proteolytic cleavage of pro-IL-1B and pro-IL-18 into their active, potent inflammatory forms. Inflammasome formation can provide this said second signal, and it is composed of one or more proinflammatory caspases; a nucleotide-binding domain, leucine-rich repeat-containing family (NLR) protein (NLRP); and the adaptor molecule ASC (apoptosis-associated speck-like protein containing a caspase-recruitment domain) [5,6].

NLRP3 is the best-described inflammasome, and murine and human data suggest that both caspase-4/5 and caspase-11 contribute to NLRP3 inflammasome activation [4]. The inflammasome-stimulatory-protein-NLRP3 encoding gene is conserved in canines [7]. Further, caspase-1/4 homologues have been identified in multiple carnivores, including canines, and caspase-1 activity has been shown in humans. There is additional evidence that hyperactivation of the NLRP3 inflammasome occurs in diverse human inflammatory diseases [8,9，^10^]. Some authors have also described the NLRP3 inflammasome in canine inflammatory diseases [11,12,13,14]. The NLRP3 inflammasome was described in canine intestinal cells; the NLRP3 inflammasome’s Casp-1, IL-1β, and IL-18 expression were evaluated [12]. This was performed to determine the effectiveness of probiotics for chronic enteritis, and found that Casp-1 and NLRP3 gene expression was lower in patients receiving probiotics compared to a control group that did not receive probiotics [12]. The results of that study suggested that the NLRP3 inflammasome or its components might be involved in the inflammation of chronic enteritis. Hirokawa et al. [13] demonstrated the increased expression of the NLRP3 and NLRP12 inflammasomes in 35 dogs with chronic enteropathy. In a naturally occurring canine model of visceral leishmaniasis (VL), glomerulonephritis manifested with complement and IgG deposition [14]. This was followed by increased light chain 3 puncta; a nucleotide-binding domain, leucine-rich repeat-containing-like receptor family; and a pyrin domain containing three associated inflammasomes, all of which are indicative of autophagosomes [14]. These authors have thus suggested that inflammasome complexes might play a role in glomerular damage during VL and autophagy. Finally, it has been reported that in ischemic myocarditis in beagles, the NLRP3 inflammasome is activated, along with elevated myocardial IL-1β and IL-18 concentrations, mediated by ROS overproduction [11].

Andrographolide is the main active ingredient in the herb *Andrographis paniculata*. A labdane diterpene, it has anti-inflammatory, antioxidant, and antineoplastic biological effects [15]. Different clinical studies have shown that andrographolide may be a useful therapeutic for different diseases, including osteoarthritis, upper-respiratory diseases, and multiple sclerosis [15]. Andrographolide may act on different targets, including the interference of transcription factors NF-κB, AP-1, and HIF-1, and signaling pathways, such as PI3K/Akt, MAPK, and JAK/STAT. It reduces the expression of several proinflammatory genes, including cyclooxygenase-2 (COX-2), IL-6, IL-8, IL-1, and inducible nitric oxide synthase (iNOS) in endothelial cells, synoviocytes, colorectal cancer cells, and leukocytes [16].

Furthermore, several studies have suggested that andrographolide can inhibit NLRP3 inflammasome activation and the ulterior release of inflammatory cytokines through different mechanisms. It has been suggested that andrographolide can modulate the signaling of inflammatory pathways and reduce reactive oxygen species production, thereby suppressing NLRP3 inflammasome activation [17]. Our hypothesis is that andrographolide decreases the expression of the NLRP3 inflammasome in canine peripheral blood mononuclear cells when stimulated with LPS and nigericin. The objective of this study is to determine andrographolide’s effect on the gene expression of selected components of the NLRP3 inflammasome, proinflammatory cytokines, and IL-1β secretion in canine peripheral blood mononuclear cells.

## 2. Materials and Methods

### 2.1. Subject Selection

Six clinically healthy, adult, mixed-breed dogs (three sterilized females, three castrated males, body weight 20–30 kg, age 3–7 years) at the Universidad Austral de Chile were selected for this study. All the animals were housed in kennels and fed adult canine pellets, with free access to water. A clinical physical examination and hemogram with complete biochemical profile were performed prior to sample collection to ensure the dogs’ good health. Each canine was sampled (20 mL) once through a sterile cephalic or jugular venipuncture, and each sample was analyzed independently. Universidad Austral de Chile’s Bioethics Committee for the Use of Animals in Biomedical Research approved all the procedures (resolution no. 551/2024).

### 2.2. Isolation of Peripheral Blood Mononuclear Cells

The peripheral blood mononuclear cells were isolated through density gradient centrifugation (Histopaque^®^-1077 solution, Merck, Darmstadt, Germany). The dogs’ peripheral blood was resuspended in PBS 1X (1:1 *v*/*v*), overlaid on half of that total volume in Histopaque^®^-1077 solution, and centrifuged at 400× *g* for 30 min at 18 °C. The peripheral blood mononuclear cells were then harvested at the interface of PBS 1X and Histopaque^®^ and washed in cold PBS 1X (300× *g*, 5 min). The red blood cells were then lysed by adding 5 mL of Lysis Buffer (eBioscience, San Diego, CA, USA) for 5 min and we stopped the reaction with 20 mL of PBS 1X, followed by centrifugation at 300× *g* (4 °C) for 10 min. The mononuclear cell purity and viability were assessed by flow cytometry (BD FACS Canto II).

### 2.3. Assessment of Cytotoxic Effect of Andrographolide by MTT Assay

To determine this, we used andrographolide (CAS 5508-58-7) with a purity of 98%, donated by HP-Ingredients (Tampa, FL, USA). To determine the cytotoxic effect of andrographolide on the canine mononuclear cells, we performed an MTT assay (3-(4,5-dimethylthiazol-2-yl)-2,5-diphenyltetrazolium bromide, Invitrogen, Waltham, MA, USA) 4 h after the incubation of the cells with andrographolide. Briefly, 4 × 10^5^ cells per well were seeded in a 96-well plate in complete RPMI 1640 medium, then treated with different concentrations of andrographolide (25, 50, and 100 µM), and incubated at 37 °C under 5% CO_2_. The control cells were only incubated with MTT, without the drug under study. Additionally, a 0.1% DMSO group was used as the vehicle control for the andrographolide. The well plate was centrifuged after incubation to remove the medium, and the cells were incubated with 120 μL of 0.5 mg/mL MTT solution for 2 h. To lyse the cells and dissolve crystal formation, the cultures were incubated with 100 μL of DMSO for 20 min. The absorbance was detected at 595 nm using a Varioskan Flash Multimode (ThermoFisher Scientific, Waltham, MA, USA) plate reader [18].

### 2.4. Real-Time Quantitative PCR Analysis

To evaluate the effects of andrographolide on inflammasome gene expression, mononuclear cells were plated onto 24-well plates (4 × 10^6^ cells/well), and pretreated with 30 or 50 µM of andrographolide for 30 min, and then stimulated with 200 ng/mL of LPS for 2.5 h at 37 °C and 5% CO_2_, and finally incubated for 1 h with nigericin (5 µM). The cell-free supernatants were collected and then stored at −80 °C for a subsequent Elisa IL-1β assay. The RNA was extracted using Trizol reagent (Invitrogen). The RNA (2 µg) was reverse transcribed using the superscript III enzyme, according to the manufacturer’s instructions (ThermoFisher Scientific). Gene amplification was performed with the RT-PCR (StepOne^TM,^ no 4376374, ThermoFisher Scientific, Waltham, MA, USA) method using Sybrgreen Master Mix (Takyon EU.UF-RSMT-B0701, Genexpress, Santiago, Chile). The sequences of the primer pairs used in this study are as follows: nlrp3 (ID: 490576): F-5′GCCCTGGGAGACTTTGGAAT3′, R-5′GTCTGGTCAGGGACTGGTT3′; asc (ID: 100856347): F-5′GGACATTGGCATGCAGGAG3′, R-5′GGTACTGCTGCTCTGACAGG3′; casp1/4 (ID:403724): F-5′CTTCGGAAAGGGCCAAATGC3′, R-5′TGTTTTCCACGAAGGCTGGT3′; IL-1β (ID:403974): F-5′AAGCCCACCCTACAGCTAGA3′, R-5′TCCTTCGACTTGAGAGGTGC3′; IL-6 (ID403985): F-5′CCTCGGCAAAATCTCTGCAC3′, R-5′CCCTCCAGTTTGGGAAGATGT3′; IL-8 (ID403850): F-5′TTGCTCTCTTGGCAGCTTTTG3′, R- 5′GGAAAGGTGTGGAGTGTGTTTT3′; and hprt1 (ID: 442945): F-5′TCATCATTACGCTGAGGATTTGGA3′, R-5′ATCCAGCAGGTCAGCAAAGAAT3′. All the primers used in this study were designed by our laboratory using Primer Blast software https://www.ncbi.nlm.nih.gov/tools/primer-blast/ (accessed on 1 July 2023). The relative expression values were normalized to the value of the housekeeping gene hprt1, and calculated based on the comparative cycle threshold Ct method (2^−ΔΔCt^ method) [19], using StepOneTM v2.3 software (Applied Biosystems, Waltham, MA, USA).

### 2.5. Enzyme-Linked Immunosorbent Assay (ELISA) Analysis

The secreted IL-1β levels were assessed using a canine IL-1β ELISA kit (ThermoFisher Scientific). The absorbance was detected at 450 nm using a Varioskan Flash Multimode (ThermoFisher Scientific) plate reader. The results were calculated using Prism 9.1.0 (GraphPad Software Inc., Boston, MA, USA), using a four-parametric-logistic (4-PL) curve fit model.

### 2.6. Statistical Analysis

All the assays represent the mean ± SEM of at least 6 independent experiments. A one-way analysis of variance (ANOVA) followed by Fisher’s LSD multiple comparison test were employed, considering a significance level of 5%. In cases where the assumptions of normality or homogeneity of variance were not met (as assessed by the Shapiro–Wilks or Brown–Forsythe tests, respectively), a Kruskal–Wallis ANOVA and Dunn’s multiple comparison test were applied. GraphPad Prism v9.1.0 (GraphPad Software, USA) was used for all the statistical analyses, and significance was established at a *p*-value < 0.05.

## 3. Results

### 3.1. Andrographolide Does Not Produce Cytotoxicity in Canine Mononuclear Cells

**Figure 1:**
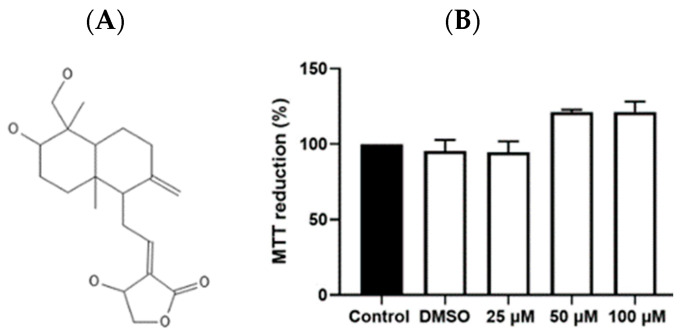
Effect of andrographolide on cell viability in canine mononuclear cells. In (**A**), the compound’s structure is depicted. The MTT assay (3-(4,5-dimethylthiazol-2-yl)-2,5-diphenyltetrazolium bromide) for 4 h is shown in (**B**). Five groups: control; DMSO 0.1%; 25, 50, and 100 µM andrographolide.

To determine the toxic effects of andrographolide on leukocyte mononuclear cells, an MTT assay was used to explore the drug-induced changes in cell viability during the incubation time of the experiment. The results indicate that exposure to andrographolide at concentrations of 25, 50, and 100 µM for 4 h did not produce a metabolic alteration or decrease compared to the control (only cells) or vehicle (DMSO 0.1%), indicating cell viability (Figure 1).

### 3.2. Andrographolide Decreases the Expression of NLRP3, Caspase-1/4, IL-1β, and IL-18

**Figure 2:**
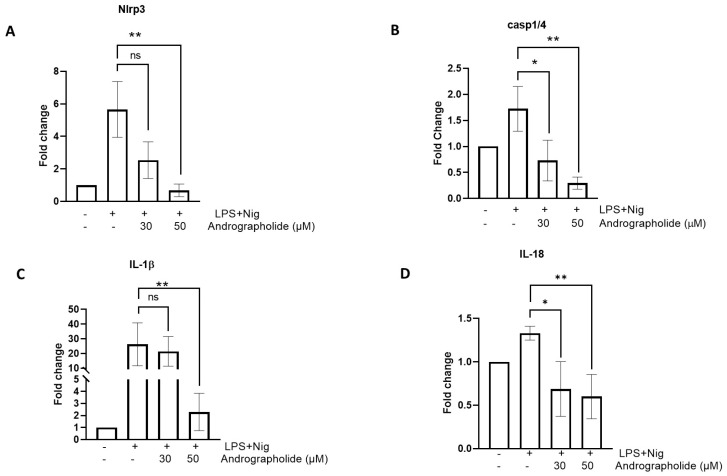
The effect of andrographolide on the expression of the inflammasome gene repertoire of canine mononuclear cells: (**A**) NLRP3; (**B**) Casp 1/4; (**C**) IL-1β; and (**D**) IL-18. Each bar represents the mean ± SD. N = 6. * *p* < 0.05; ** *p* < 0.01.

A real-time PCR analysis revealed that andrographolide produces a significant decrease in the gene expression of some components of the inflammasome. As shown in Figure 2A, NLRP3 mRNA expression levels are significantly higher in the cells stimulated with LPS plus nigericin. However, in the presence of 50 µM andrographolide, the NLRP3 mRNA expression levels decrease significantly. In turn, this same phenomenon is observed with caspase-1/4, in which the caspase-1/4 mRNA expression values increase when stimulated with LPS plus nigericin, and decrease significantly in the presence of 50 µM andrographolide (Figure 2B). In the case of IL-1β, treatment with 30 µM andrographolide does not significantly decrease its expression. However, a 50 µM treatment does produce a significant downregulation of the *IL-1β* gene (Figure 2C). In turn, LPS plus nigericin produces an increase in IL-18 expression, but in the presence of andrographolide there is a statistically significant decrease in the expression of this cytokine (Figure 2D).

### 3.3. Andrographolide Decreases the Expression of ProInflammatory Cytokines

**Figure 3:**
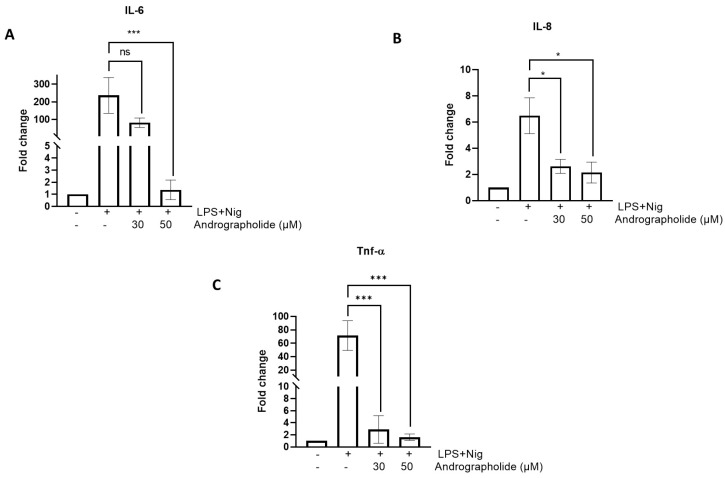
Effect of andrographolide on the expression of proinflammatory cytokines in canine mononuclear cells: (**A**) IL-6; (**B**) IL-8; and (**C**) TNF-α. Each bar represents the mean ± SD. N = 6. * *p* < 0.05; *** *p* < 0.001.

Figure 3 illustrates that andrographolide produces a decrease in proinflammatory cytokine expression. As seen in Figure 3A, LPS plus nigericin markedly increases the relative expression of IL-6; 30 µM andrographolide produces a decrease in gene expression, but this is not significant. However, treatment with 50 µM andrographolide produces a significant decrease in the relative IL-6 expression (*p* < 0.01). With respect to IL-8 (Figure 3B), the LPS plus nigericin stimulus produces an increase in the relative expression of the cytokine; however, it is not of the same magnitude as that shown by IL-6. Amounts of 30 and 50 µM andrographolide, respectively, induce a significant relative downregulation of the IL-8 gene (*p* < 0.05). LPS plus nigericin stimulation produces an increase in the relative TNF-a gene expression (Figure 3C) in the same manner as that for IL-6. Treatment with 30 and 50 µM andrographolide significantly reduce TNF-a expression (*p* < 0.01).

### 3.4. Andrographolide Decreases IL-1β Secretion

**Figure 4:**
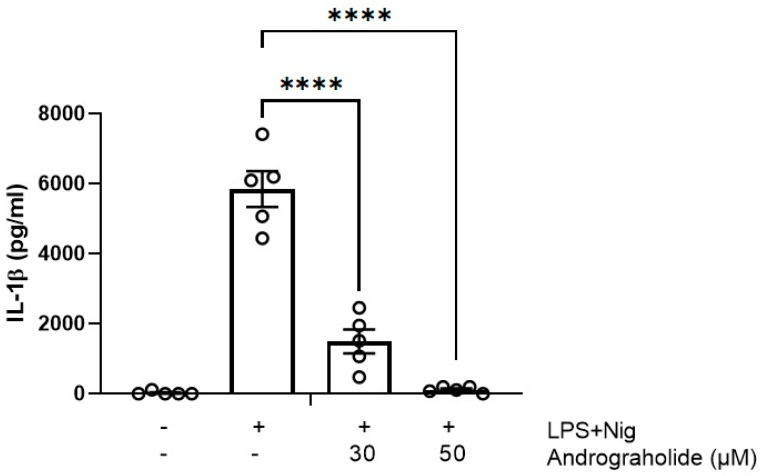
Effect of andrographolide on IL-1β secretion in canine mononuclear cells. Each bar represents the mean ± SD. N = 6. **** *p* < 0.0001.

LPS plus nigericin produced a marked increase in IL-1β secretion (Figure 4), but in the presence of 30 µM and 50 µM andrographolide, the IL-1β levels dropped significantly (*p* < 0.001), even reaching concentrations of 0 pg/mL after the 50 μM treatments.

## 4. Discussion

In this manuscript, we describe the effect of andrographolide on the expression of selected component genes of the NLRP3 inflammasome and several proinflammatory cytokines, as well as IL-β secretion by canine peripheral blood mononuclear cells stimulated with LPS plus nigericin. The initial results reveal that the mononuclear cells, when stimulated, exhibit an increase in the genes that constitute the inflammasome complex, specifically NLRP3 and caspase-1/4. Inflammasomes are regulated by caspases that function upstream or downstream of this protein complex, including caspase-1, murine Casp-11, or human Casp-4 and -5 [20]. These enzymes usually all have an N-terminal caspase activation and recruitment domain (CARD) that is fused to an enzymatic domain [21]. However, dogs, which belong to the order Carnivora, deviate from this pattern. They lack the gene that encodes Casp-1 [22,23]. Instead, in dogs, a Casp-1-like CARD is fused to a second CARD and an enzymatic domain, both of which resemble human Casp-4. These *Canis lupus familiaris* Casp-1/4 proteins exhibit all the activities of Casp-1, including pro-IL-1β cleavage activity [21]. The same authors have suggested that canine Casp-1/4 proteins function similarly to Casp-1 homologues in mice and humans. This observation aligns with our results, as domestic canine mononuclear cells stimulated with LPS plus nigericin exhibited a significant increase in the caspase-1/4 transcript, along with enhanced IL-1β expression and secretion. Other authors have indicated that P2X7 may also play an important role in IL-1β-dependent processes in dog monocytes [24].

Andrographolide’s inhibitory effect on inflammasome activation has been explored previously [17]. Andrographolide has been proposed as a multitarget drug with anti-inflammatory, antioxidant, and antineoplastic effects on different cell lines [15]. This molecule has been shown to reduce proinflammatory gene expression, including cyclooxygenase-2 (COX-2), IL-6, IL-8, IL-1β, and inducible nitric oxide synthase (iNOS) [16]. Further, in models of Freund’s adjuvant-induced complete arthritis or collagen-induced arthritis in rodents, andrographolide has decreased the clinical score of arthritis and joint damage, as well as NO and TNF-α production [25]. It has been demonstrated that the intravenous administration of andrographolide to rodents reduces the proinflammatory and hemodynamic effects of LPS [8]. Andrographolide also protects against LPS-induced acute lung injury by reducing myeloperoxidase (MPO) activity; neutrophil and macrophage relative concentrations; and TNF-, IL-6, and IL-1β in mice bronchoalveolar lavage fluid [26]. In a different study on monosodium urate and LPS-activated murine macrophages or monocytes, andrographolide dose-dependently decreased IL-1β and caspase-1 [27]. In our study, we found that andrographolide resulted in a significant decrease in the expression of NLRP3, caspase-1/4, IL1-β, and IL-18 in canine monocyte peripheral blood. Therefore, andrographolide reduces the expression of genes associated with the inflammasome complex and leads to a significant reduction in IL-1β secretion. It also decreases proinflammatory cytokines, such as IL-6, IL-8, and TNF-α, as described in the aforementioned studies. These findings suggest that andrographolide may serve as a wide-acting anti-inflammatory agent.

NF-κB is central to the pathogenesis of inflammation, and several drugs in use for human inflammatory diseases focus on inhibiting NF-κB activation [28]. Several components of the immune system are regulated by this transcription factor, including proinflammatory cytokines, chemokines, adhesion molecules, and inducible enzymes such as COX2 and iNOS, in addition to other proteins controlling specific immune responses [29]. Thus, the dysregulation of NF-κB may well lead to inflammatory and autoimmune diseases. Andrographolide can attenuate inflammation by the direct inhibition of NF-κB activation. It does this by inhibiting the p50 and p65 heterodimer subunits that make up this transcription factor, thus blocking the binding of the NF-κB oligonucleotide to nuclear proteins [30,31]. Although we did not directly assess the effect of andrographolide on NF-κB in this study, we did observe a significant inhibitory effect of andrographolide on the expression of the proinflammatory cytokines that are regulated by NF-κB, suggesting a possible indirect inhibiting effect of andrographolide on NF-κB. Furthermore, andrographolide exerts its inhibitory effects on the NLRP3 inflammasome through multiple mechanisms. It has been shown to interfere with the assembly of the NLRP3 complex by modulating mitochondrial reactive oxygen species (ROS) production and reducing mitochondrial DNA release [32,33]. Additionally, andrographolide inhibits NF-κB signaling, a critical pathway in the priming step of NLRP3 activation. By dampening these upstream signals, andrographolide effectively reduces the secretion of IL-1β and IL-18, thereby mitigating inflammation [17].

Andrographolide has shown significant potential in veterinary medicine, especially in canine health [34,35]. Its anti-inflammatory and immunomodulatory properties make it a promising candidate for the treatment of various inflammatory conditions in dogs, in which NLRP3 and proinflammatory cytokines (mainly IL1-β and IL-18) are involved. The studies described above have shown that andrographolide can modulate immune responses, reduce oxidative stress and inhibit proinflammatory cytokines. This could be crucial for the possible management of diseases, such as canine atopic dermatitis, arthritis, and other chronic inflammatory conditions in this species. Despite promising findings in human medicine, larger clinical trials are needed to fully establish the efficacy and safety of andrographolide in dogs. Current preclinical studies highlight its potential, but a thorough understanding of its pharmacokinetics, optimal dosing, and long-term effects is essential for its widespread application in veterinary medicine. The results obtained in this study may justify further research on andrographolide, and its relationship with the NLRP3 inflammasome, as a possible standard treatment option for inflammatory pathologies in dogs.

## 5. Conclusions

These results indicate that andrographolide can interfere with the activation and function of the NLRP3 inflammasome, resulting in a reduction in the inflammatory response. Research on andrographolide is certainly ongoing, and further studies are needed to fully understand its mechanisms of action and therapeutic potential in relation to the NLRP3 inflammasome in dogs.

## Data Availability

The data presented in this study are available on request from the corresponding author.
